# Complete mitochondrial genome of *Lelecella limenitoides* (Lepidoptera: Nymphalidae: Apaturinae) and phylogenetic implication

**DOI:** 10.1080/23802359.2020.1730268

**Published:** 2020-02-27

**Authors:** Li Jun Fang, Shao Li Mao, Ya Lin Zhang

**Affiliations:** a Shaanxi Engineering Research Centre for Conservation and Utilization of Botanical Resources, Xi’an Botanical Garden of Shaanxi Province (Institute of Botany of Shaanxi Province), Xi’an, China;; b Key Laboratory of Plant Protection Resources and Pest Management, Ministry of Education, Entomological Museum, Northwest A&F University, Shaanxi, China

**Keywords:** *Lelecella limenitoides*, Apaturinae, mitochondrial genome, phylogeny

## Abstract

The length of *Lelecella limenitoides* complete mitogenome was 15,203 bp and contained the typical gene arrangement, base composition, and codon usage found in other related species. The overall base composition exhibited obvious anti-G (7.5%) and AT bias (81.6%). The initiation codons of all PCGs were typical ATN (ATA/ATG/ATT), and the termination codons were TAA, TAG, or incomplete stop codon T—. All tRNAs could be folded into typical cloverleaf secondary structures, except tRNA^Ser^ (AGN). Phylogenetic analysis showed that *L. limenitoides* was clustered with the clade of *Sasakia*, *Euripus,* and *Apatura*.

Apaturinae is a subfamily of Nymphalidae with 16 genera about 91 species globally, 14 genera 47 species are distributed in China. In present, the mitogenomes of nine species belonging to six genera had been sequenced and deposited in GenBank (Wang et al. 2012, 2013, 2016, 2015; Zhang et al. 2012; Cao et al. 2013; Chen et al. 2013; Xuan et al. 2016). In this study, the species *Lelecella limenitoides* was sequenced using Illumina Hiseq 2500 platform and assembled using MitoZ (Meng et al. 2019). The whole mitochondrial genome sequence was annotated using the software Geneious v2020.0.4 (Kearse et al. 2012). The tRNA genes and their potential structures were predicted using the online software MITOS (Bernt et al. 2013). The specimen was collected from Xi’an (Shaanxi, China) (E108°95.971′, N33°91.401′) in 2019 and was deposited in Butterfly specimen room of Xi’an Botanical Garden of Shaanxi Province (no. 201905041).

**Figure 1:**
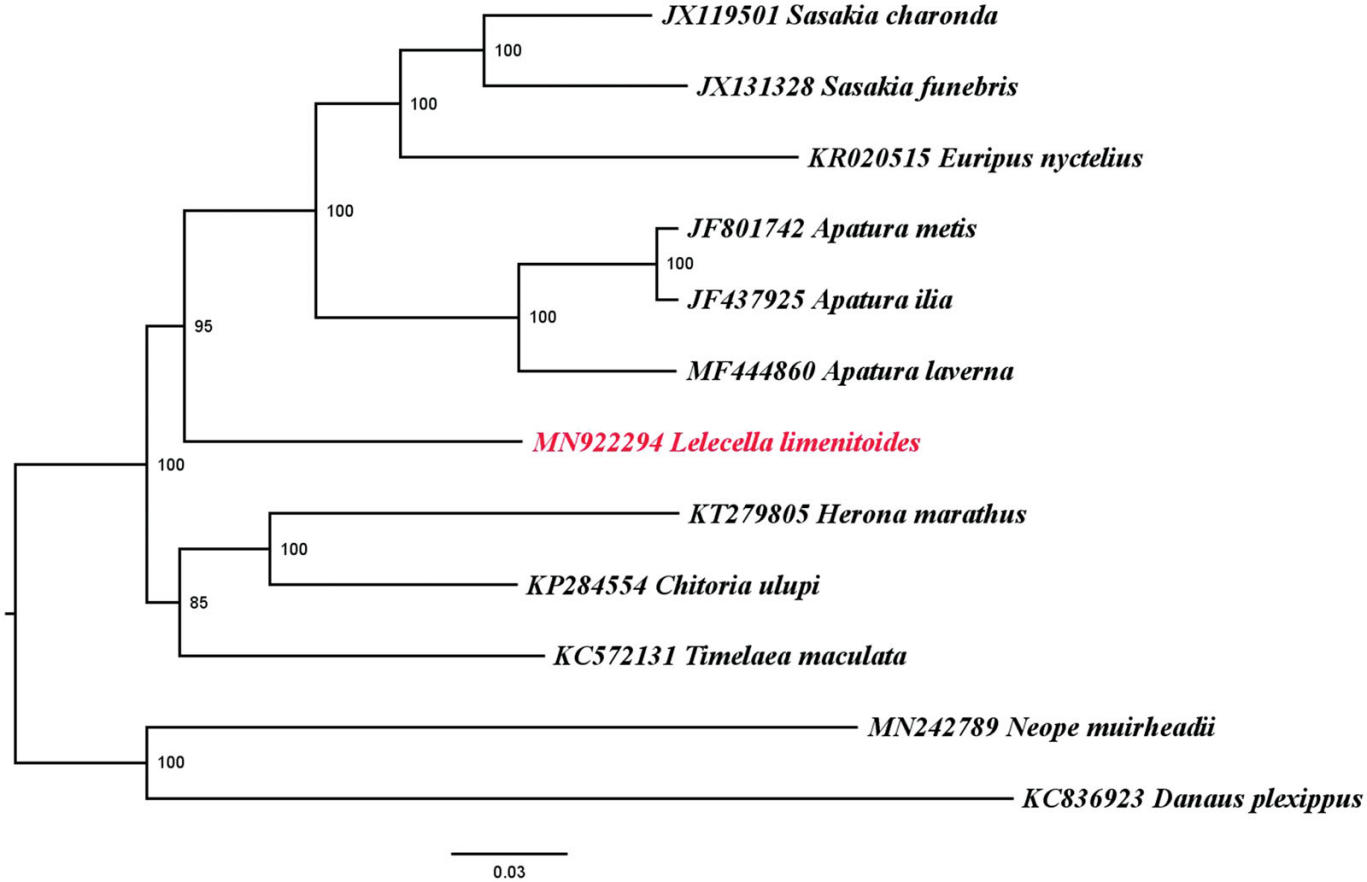
Phylogenetic reconstruction of the Apaturinae using mitochondrial PCGs and rRNA of the concatenated dataset.

The complete mitogenome of *L. limenitoides* is 15,203 bp in length and has been deposited in GenBank (accession no. MN922294). It consists of 13 protein-coding genes, 22 tRNA genes, two rRNA genes, and one control region. Gene content and arrangement are identical with other Lepidoptera mitogenome. Mitochondrial genes are separated by a total of 113 bp of intergenic spacer sequences, which are spread over 12 regions and the longest one locates between tRNA^Gln^ and ND2. There are 13 overlaps with all of 41 bp. The overall base composition of the whole mitochondrial genome is 39.8% A, 41.8% T, 10.9% C, and 7.5% G, obvious anti-G and AT bias (81.6%).

The initiation codons of all PCGs are typical ATN (COII, ATP6, COIII, ND1, ND4, ND4L, and Cytb with ATG, ND2, ATP8, ND5, ND6 with ATT; ND3 with ATA). Nine protein genes (ND2, COI, ATP8, ATP6, COIII, ND4L, ND6, Cytb, and ND1) use TAA as the termination codons, and only one gene (ND3) is stopped with TAG. COII, ND5, and ND4 have an incomplete stop codon T—. The length of tRNA genes ranked from 61 bp (tRNA^Ser (AGN)^) to 73 bp (tRNA^Leu (CUN)^). All tRNAs exhibit typical cloverleaf secondary structures, except tRNA^Ser (AGN)^ lacks the DHU arm, a feature generally present in all Lepidoptera insects as well as in other metazoan mitogenomes (Lavrov et al. 2000). The length of 12S rRNA and 16S rRNA are 713 bp and 1324 bp respectively, separated by tRNA^Val^. The control region of *L. limenitoides* mitogenome is located at the conserved position between 12S rDNA and tRNA^Ile^-tRNA^Gln^-tRNA^Met^ gene cluster and 448 bp in length.

Phylogenetic analysis of Apaturinae species was performed on the concatenated datasets of 13 PCGs and two rRNA genes by IQ-tree (Nguyen et al. 2015). Phylogenetic topology of the genera was congruent with previous study (Wang et al. 2017). The new sequenced species *L. limenitoides* clustered with the genera of *Sasakia*, *Euripus*, and *Apatura* and then clustered together with the clade of *Herona*, *Chitoria*, and *Timelaea.*
